# Nickel Based Electrospun Materials with Tuned Morphology and Composition

**DOI:** 10.3390/nano6120236

**Published:** 2016-12-06

**Authors:** Giorgio Ercolano, Filippo Farina, Sara Cavaliere, Deborah J. Jones, Jacques Rozière

**Affiliations:** Institut Charles Gerhardt de Montpellier, Agrégats Interfaces et Matériaux pour l’Energie, UMR 5253 CNRS, Université de Montpellier, 34095 Montpellier CEDEX 5, France; giorgio.ercolano@umontpellier.fr (G.E.); filippo.farina@umontpellier.fr (F.F.); deborah.jones@umontpellier.fr (D.J.J.); jacques.roziere@umontpellier.fr (J.R.)

**Keywords:** electrospinning, nanofibers, nickel, nickel oxide, nickel nitride

## Abstract

Nickel is set to play a crucial role to substitute the less-abundant platinum in clean electrochemical energy conversion and storage devices and catalysis. The controlled design of Ni nanomaterials is essential to fine-tune their properties to match these applications. A systematic study of electrospinning and thermal post-treatment parameters has been performed to synthesize Ni materials and tune their morphology (fibers, ribbons, and sponge-like structures) and composition (metallic Ni, NiO, Ni/C, Ni_3_N and their combinations). The obtained Ni-based spun materials have been characterized by scanning and transmission electron microscopy, X-ray diffraction and thermogravimetric analysis. The possibility of upscaling and the versatility of electrospinning open the way to large-scale production of Ni nanostructures, as well as bi- and multi-metal systems for widened applications.

## 1. Introduction

Considering the low abundance of platinum, the research interest in common metals to replace it for electrochemical and catalytic reactions is renewed. In this context, nickel based materials (sharing the same group of the periodic table with Pt) play a crucial role in hydrogen production, as well as in several clean energy conversion and storage devices. Metallic nickel, as well as its oxides and nitrides, due to their great (electro) catalytic activity and low cost, are widely used to generate H_2_ by reforming reactions [1,2], borohydride decomposition [3], water electrolysis [4,5,6] and splitting [7]. Nickel and its compounds are also employed as electrocatalysts, co-catalysts or catalyst supports in fuel cells operating in various conditions and temperatures, including solid oxide [8], proton exchange membrane [9,10,11,12], alkaline [13,14], and microbial fuel cells [15]. Nickel materials also catalyze other environmentally relevant reactions including the electrochemical conversion of CO_2_ [16,17]. Of particular interest is the use of nickel nanowires as the starting point for the synthesis of core@shell nickel@platinum one-dimensional (1D) electrocatalysts, using a simple galvanic displacement reaction, which was able to surpass the U.S. Department of Energy target of oxygen reduction mass activity for proton exchange membrane fuel cells (PEMFC) [9,18,19], which provided one source of motivation for this work.

To further improve the physico-chemical and (electro) catalytic properties of nickel, the preparation of nickel nanofibers with a three-dimensional (3D) network structure is of particular interest. Indeed, not only their considerable surface to volume ratio could be exploited, but also their porous 3D architecture could be beneficial for electrochemical and catalytic reactions. 

Ni-based nanowires have been synthesized by several chemical and physical routes, including electrodeposition on 1D materials (e.g., carbon nanotubes [20]) or templates (e.g., polycarbonate membranes [21]), electroless plating [22], chemical vapor deposition [23], microwave-assisted synthesis [24], solvothermal [25] and wet chemical processes [26]. Ni and NiO nanowires have been also prepared by electrospinning [27,28,29,30,31]. This technique presents several advantages compared to the other physico-chemical synthesis routes cited above: efficiency, reproducibility, high yield, simplicity, low-cost, and high versatility [32]. The latter concerns not only the chemical compositions, but also the wide range of morphologies and architectures achievable. Furthermore, the preparation of electrospun materials is easily up-scalable through the availability of setups including multiple needle or needle-less electrospinning. In the recent papers exploring electrospinning of Ni-based materials, however, no systematic study has been made of the parameters allowing targeted compositions and morphologies to be achieved. 

The rational design of Ni-based nanomaterials with controlled morphologies and compositions is crucial to fine-tune their properties in view of specific catalytic and electrocatalytic applications. In the present work, we describe a general route using electrospinning to obtain nickel-based 1D nanomaterials. Several synthesis parameters have been investigated, including the metal precursor, solvent and carrier polymer, as well as the thermal post-treatments performed (number of steps, gas atmosphere, and temperature). We demonstrate a range of strategies to tune the morphology (nanofibers, nanoribbons, sponge-like structures) and composition (Ni, NiO, Ni_3_N, Ni/C as well as their combinations), thus giving a means of obtaining the appropriate materials for the targeted application. Electrospinning is straightforward and up-scalable, allowing for large-scale production and enabling new and industrially relevant opportunities for the manufactured materials. 

## 2. Results and Discussion

### 2.1. Morphological and Structural Characterization of the Ni-Based Electrospun Materials

**Figure 1:**
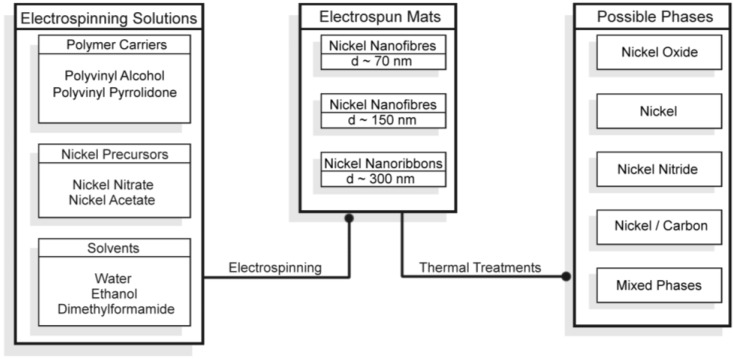
Schematic representation of the synthesis routes leading to Ni-based one-dimensional (1D) materials by electrospinning and thermal treatments.

For the preparation of nickel-based electrospun nanofibers, the conventional approach consists of two main steps: (1) the electrospinning of a metal precursor with a carrier polymer (to obtain salt/polymer 1D structures) and (2) the calcination in air of the as-spun materials (to remove the polymer and obtain metal oxide fibers); if metallic nickel is the desired phase, the calcination is followed by the reduction of the oxide to metal fibers. In this work, we started from this route and first investigated the electrospinning solution parameters, namely the metal precursors (nickel acetate and nitrate), the carrier polymers (polyvinylpyrrolidone—PVP—and polyvinylalcohol—PVA—and the solvents (water, ethanol and ethanol with addiction of water or dimethylformamide—DMF). Furthermore, we modified the thermal treatments adding or removing intermediate steps and varying the gas atmospheres. A schematic representation of the general approach we used is depicted in Figure 1.

The electrospinning process is the deciding factor in tuning the morphology: the 1D materials shape (fibers, ribbons or a combination of the two) and the diameter distribution depend on this step; further minor morphological modifications and the final crystalline phases are controlled by the successive thermal treatments.

#### 2.1.1. Morphological Differences of Different Electrospinning Based NiO Synthesis

At first, we focused on the final morphology of 1D NiO obtained using various electrospinning procedures to produce polymer/nickel precursor mats followed by a conventional calcination in air at 600 °C to remove the carrier polymer and oxidize the nickel salts to nickel oxide. The concentration and spinning parameters shown were chosen after careful optimization aimed at maximizing the suitability and reproducibility of the experiment and the uniformity of the spun mats obtained.

**Figure 2:**
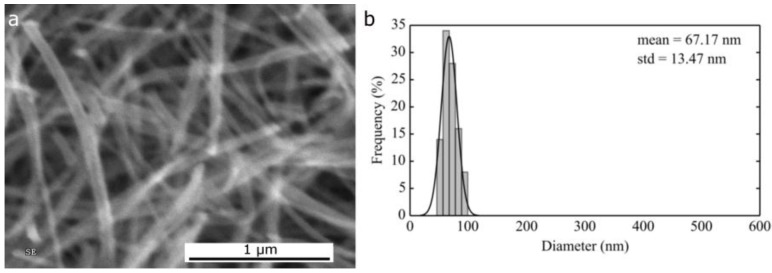
(**a**) Scanning electron microscopy (SEM) micrograph and (**b**) corresponding histogram of fiber size distribution of NiO fibers obtained from 12 wt % polyvinylalcohol and 12 wt % nickel acetate in H_2_O.

The NiO nanofibers in Figure 2 have an average diameter of 67 nm (σ = 13.5 nm) and were obtained by spinning an aqueous solution of PVA and nickel acetate; these results are in line with those obtained by a similar synthesis [33] and smaller than the NiO nanofibers with an average of 135 nm previously reported for electrospun PVA/Ni(NO_3_)_2_ [28]. This route proved to be extremely slow as flow rates larger than 0.2 mL/h could not be applied. Due to the nature of the polymer, specifically its limited solubility in ethanol and DMF, this composition could not be investigated in the formulation of alternative electrospinning solutions.

**Figure 3:**
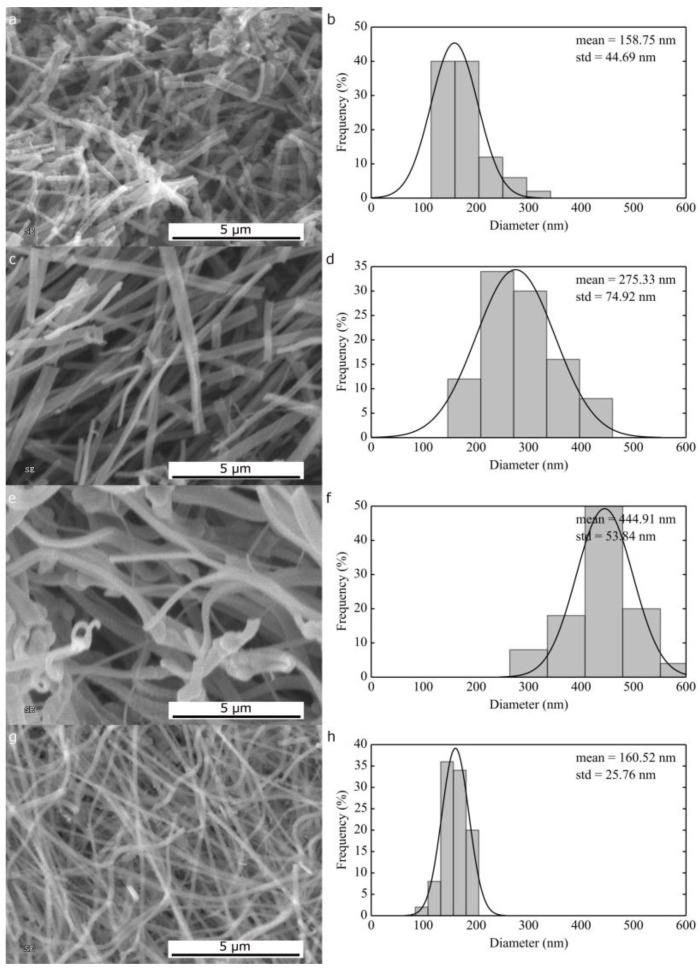
SEM micrographs and corresponding histograms of size distribution of NiO electrospun in the same conditions as 9 (**a**,**b**), 10 (**c**,**d**), 11 wt % (**e**,**f**) polyvinylpyrrolidone and nickel acetate in ethanol (**a**,**b**) and in ethanol:dimethylformamide (7:3 vol.) (**g**,**h**).

The morphology of 1D NiO materials obtained spinning a solution of PVP and nickel acetate in pure ethanol is ribbon-like (Figure 3a,c,e) and extremely dependent on the solution concentration: small increases in carrier polymer concentration from 9 wt % to 10 wt % changed the average dimension from 159 nm (σ = 45 nm) to 275 nm (σ = 75 nm), in agreement with dynamic viscosity values (Table 1). The tendency to form ribbons can be associated with the rapid evaporation of the solvent that can produce a dry polymer skin around the electrospun jet; these tubes then collapse under electro-dynamic forces once the target is reached (especially in the case of rotating drums) forming continuous ribbons [34,35]. 

A further increase in concentration from 10 wt % to 11 wt % led to instability during the spinning and to the formation of secondary jets together with an increase in ribbon average dimension (Figure 3e,f): thinner fibers can be observed amidst the ribbons of 445 nm (σ = 54 nm) [35]. The large σ values observed for ribbon-like structures is related to a variation in the observable lateral dimension as a function of the angle between the flat side of the ribbon and the SEM point of view; what is measured is the projected surface dimension that does not always coincide with the real dimension.

Replacing pure ethanol with a mixture of ethanol/DMF (7:3 vol.) in the electrospinning solution, solid skin formation is prevented by the presence of the higher boiling point solvent and the 1D morphology produced is fibrous and not ribbon-like; thick nanofibers with average diameter of 160 nm (σ = 25 nm) were obtained (Figure 3g,h).

The change of nickel salt from acetate to nitrate leads to the impossibility of using pure ethanol as solvent for the electrospinning solution; a mixture of water or DMF and ethanol is necessary in order to prevent polymer precipitation.

**Figure 4:**
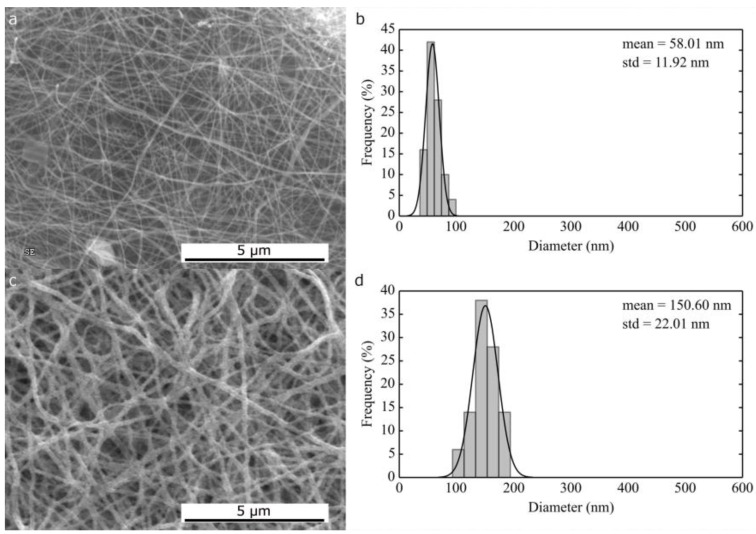
SEM micrographs and corresponding histograms of fiber size distribution of NiO from water/ethanol (**a**,**b**) and dimethylformamide/ethanol (**c**,**d**).

In addition, in this case, the presence of a higher boiling point solvent in the solution favoured formation of fibers, rather than ribbons (Figure 4). A large difference is found in the average diameter of the NiO fibers: those obtained from 11 wt % PVP and 11 wt % nickel nitrate in H_2_O/ethanol (1:1 wt ratio) have an average diameter of 58 nm (σ = 12 nm), while the fibers obtained from 9 wt % PVP and 9 wt % nickel nitrate in DMF/ethanol (1:1 wt ratio) have an average diameter of 151 nm (σ = 22 nm). In this case, it is evident that the nature of the solvent has a larger impact on the materials obtained than the concentration of the solution. Surprisingly, a water-based solution led to the formation of fibers three times smaller than the DMF counterpart, despite the higher salt and polymer concentration. This phenomenon can be attributed to the different rheological properties of the two electrospinning solutions. The dynamic viscosity of the water/ethanol solution has been found to be higher than that using DMF/ethanol (0.130 Pa·s vs. 0.071 Pa·s), which alone cannot explain the lower diameter obtained with the first [34]. A major role can be played by the higher conductivity of the water based solution, mainly due to higher ion content, as well by its higher surface tension.

#### 2.1.2. Thermal Treatment Effect on Crystalline Structure and Grain Morphology

NiO is not the only crystalline phase that can be obtained after thermal treatment of polymer/Ni salt fibers. Nevertheless, it is usually the first stage of a more complex multi-step process when metallic nickel or nickel nitrides are the desired phases. While NiO is obtained by direct calcination in air of the as-spun materials, other phases can be obtained by further thermal treatments in various atmospheres. Furthermore, tuning the electrospinning process is not the only way to change the final morphology. In particular, temperature and duration of the thermal treatments that follows the calcination play a crucial role in determining the final morphology of the nanomaterial.

**Figure 5:**
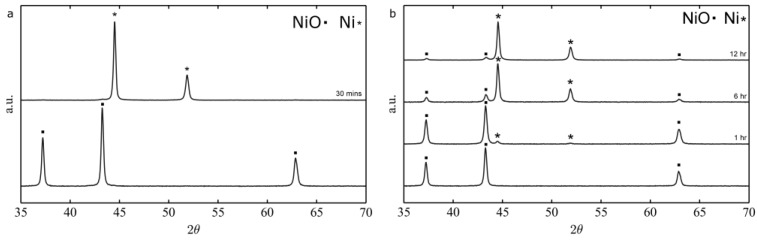
X-ray diffraction (XRD) diffractograms of the conversion of NiO to Ni fibers at 250 °C in pure hydrogen (**a**) vs. H_2_/Ar; and (**b**) Joint Committee on Powder Diffraction Standards (JCPDS) reference: NiO 96-101-0094, Ni 96-151-2527.

Hydrogen mediated nickel oxide reduction to metallic nickel is a well-known process [36,37,38,39] that has been successfully applied to electrospun nanofibers [28]. When pure hydrogen atmosphere is used, a temperature as low as 250 °C and a 30 min time are sufficient to fully convert pure NiO fibers into Ni. However, when a mixture of hydrogen and argon (5% hydrogen) is used at 250 °C, even times as long as 12 h are not sufficient to completely reduce NiO, as demonstrated by the XRD diffractograms depicted in Figure 5.

**Figure 6:**
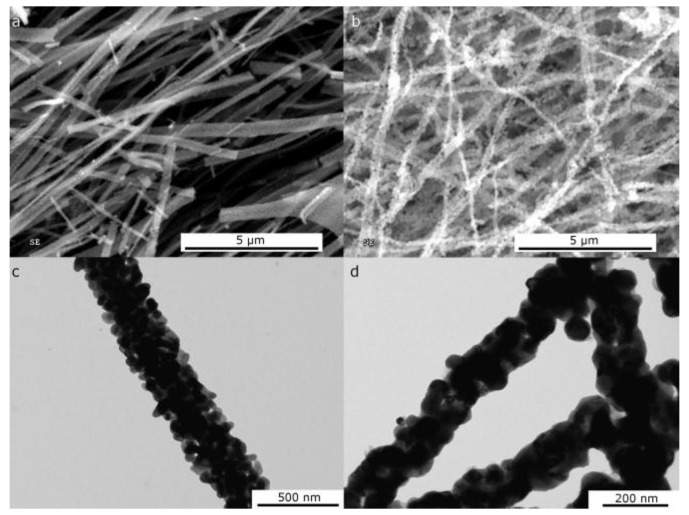
SEM micrographs of NiO ribbons (obtained from polyvinylpyrrolidone (PVP) and nickel acetate in ethanol) and transmission electron microscope (TEM) micrographs of NiO nanofibers (obtained from PVP and nickel acetate in DMF/ethanol) before (**a**,**c**) and after reduction at 250 °C in H_2_ (**b**,**d**).

A common result of the reduction process is a slight increase of the crystallite grain size as presented both in the SEM micrograph of the ribbons and in the TEM micrograph of nanofibers before and after the reduction step (Figure 6).

**Figure 7:**
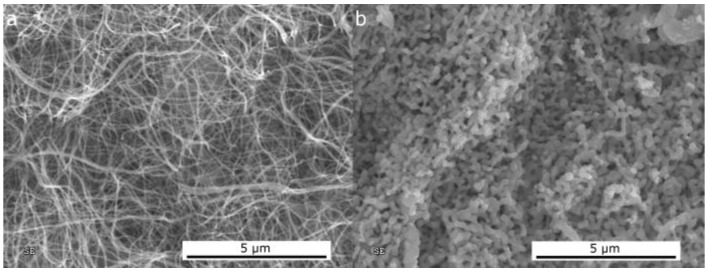
SEM micrographs of electrospun Ni (from polyvinylalcohol and nickel acetate in water) obtained after reduction in H_2_ at 250 °C (**a**) and 400 °C (**b**).

Loosely connected ribbons or fibers can be converted into an interconnected sponge-like nanostructure by increasing the reduction temperature: the SEM image in Figure 7 shows the same NiO nanofibers reduced at 250 °C and 400 °C.

**Figure 8:**
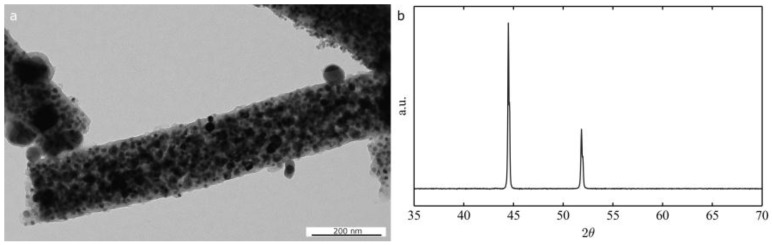
TEM micrograph of Ni/C composite fibers (obtained from PVP and nickel acetate in DMF/ethanol at 700 °C under nitrogen (**a**) and the corresponding XRD diffractogram (**b**) JCPDS reference: Ni 96-151-2527.

An alternative strategy to the calcination followed by the reduction process described above is the thermal decomposition of a solution of the polymer and the nickel salt at high temperature (700 °C) in inert atmosphere that can directly furnish metallic nickel crystals. This process tends to produce hybrid carbon/nickel materials that can be potentially used as catalysts for hydrocarbon hydrocracking [40] and supercapacitors [41]. From the TEM micrograph in Figure 8a, it is clear that the fibers are constituted of nickel grains in a carbonaceous matrix. The XRD in Figure 8b confirmed that the only crystalline phase present is pure metallic nickel. From thermogravimetric analysis in air, a carbon content of 25 wt % was calculated for the PVP derived fibers. This value can be reduced by replacing PVP with other carrier polymers. As an example, we can cite polyvinylbutyral, leading to a small residual carbon contamination (below 1 wt %).

**Figure 9:**
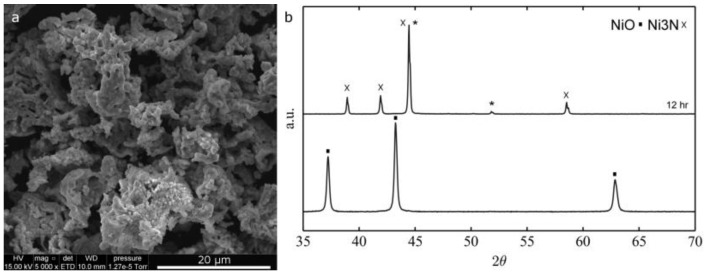
SEM micrograph of a sponge-like Ni_3_N structure (obtained from NiO nanofibers derived from PVP and nickel acetate in DMF/ethanol) (**a**) and the corresponding XRD diffractogram (**b**) before and after the two-step complete conversion in NH_3_. JCPDS reference: Ni_3_N 00-010-280, Ni 96-151-2527.

An alternative nickel crystalline phase that received attention for its application for examples in Li-ion batteries, supercapacitors and solar cells is nickel nitride [42,43]. Changing the reducing atmosphere from hydrogen to ammonia and controlling the reaction temperature, it is possible to obtain nickel nitride from nickel oxide or metallic nickel. At low temperatures, the direct conversion in NH_3_ of nickel oxide to nitride is not possible [44]. On the other hand, at a high temperature, the Ni_3_N obtained treating the oxide in ammonia is not stable and spontaneously reduces to Ni(0). It is thus possible to obtain Ni_3_N from NiO by modulating the temperature of the ammonia treatment: first, the oxide is reduced to nickel metal at high temperature (650 °C), and then it is converted to the nitride at low temperature (250 °C) (Figure 9b). The high temperature reduction tends to profoundly change the morphology and the fibrous structure is lost (Figure 9a), giving rise to a sponge-like Ni_3_N structure.

**Figure 10:**
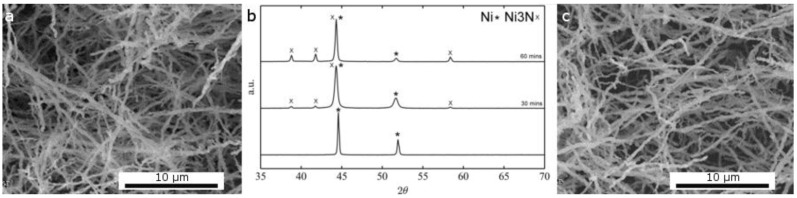
SEM of Ni fibers before (**a**) and after thermal treatment under NH_3_ (**c**) and XRD diffractogram of fibers with different degrees of conversion to the nitride phase (**b**) JCPDS reference: Ni_3_N 00-010-280, Ni 96-151-2527.

Alternatively, metallic nickel fibers can be converted to nickel nitride in ammonia at 250 °C. In this case, time can be used to control the degree of conversion without changes to the original morphology: nickel nitride or nickel/nickel nitride nanofibers can thus be obtained (Figure 10).

### 2.2. Application of Nickel Electrospun Nanofibers in Fuel Cells

Core@shell nickel@platinum nanofibres have demonstrated being promising electrocatalysts for PEMFC [9,18,19]. They can be prepared by the straightforward and efficient galvanic displacement based on the exchange of peripheral metal ions with ions from another metal with a higher reduction potential. This opens the possibility of forming a thin platinum film around nanofibres fabricated using less expensive, more abundant metals than platinum such as nickel. The 2D morphology of the electrocatalyst films has been shown to produce 10 fold higher surface activity (minimizing unused noble metal) and better durability than conventional Pt/C electrocatalysts (lowering electrochemical Ostwald ripening) [45].

**Figure 11:**
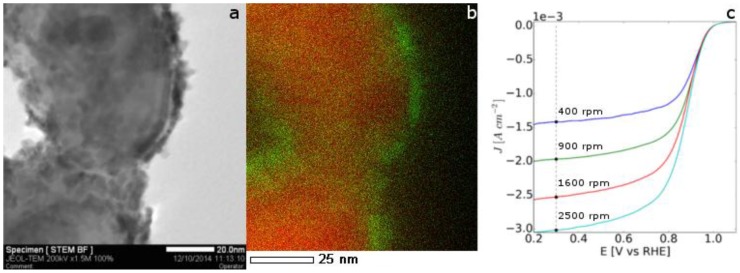
STEM image of platinum on nickel nanofibres after the galvanic displacement (**a**), overlay of the EDX maps of Ni (**red**) and Pt (**green**) (**b**) and oxygen reduction reaction (ORR) at 5 mV/s in 0.1 M HClO_4_ saturated with O_2_ at 400, 900, 1600, 2500 RPM (**c**).

In this work, the Pt/Ni galvanic displacement has been performed [46] onto nickel fibres obtained by electrospinning solutions of PVP and nickel acetate in ethanol, followed by calcination and reduction steps (see Figure 6d). In the STEM image and the EDX maps of the obtained Ni@Pt 1D nanomaterials (Figure 11a,b) platinum presents an island-like morphology, demonstrating the efficient replacement of surface Ni atoms by Pt. Furthermore, the nickel core does not appear to be damaged or etched. 

Electrochemical characterisation has been performed on these core@shell electrocatalysts in acidic medium. The electrochemical surface area calculated from the cyclic voltammetry (not shown here) was 21 m^2^/g in agreement with metal islands instead of separated nanoparticles. No nickel peaks are detected, showing that the inner metal is not exposed as a contiguous Pt coverage has been achieved. The activity of the Pt/Ni nanofibres towards the oxygen reduction reaction (ORR) was evaluated in HClO_4_. From the ORR currents reported in Figure 11c, a mass activity at 0.9 V/RHE of 42 A/g can be calculated.

## 3. Materials and Methods 

### 3.1. Materials

Nickel(II) nitrate hexahydrate (Sigma-Aldrich St. Louis, MO, USA, puriss, p.a. > 98.5%) and nickel(II) acetate tetrahydrate (Aldrich, St. Louis, MO, USA, purum, p.a. > 99.0%) were used as nickel precursors salts. Polyvinylalcohol (Aldrich, Milwakee WI, USA, 98%–99% hydrolized M_w_ 85,000–146,000), polyvinylbutyral (Butvar B98, Sigma, St. Louis, MO, USA) and polyvinylpyrrolidone (Aldrich, Steinheim, Germany, M_w_ ~ 1,300,000) were used as carrier polymers. MilliQ water (18 MΩ), *N*,*N*-dimethylformamide (Sigma-Aldrich Chromasolv^®^Plus for HPLC > 99.9%, Irvine, UK) and ethanol (Sigma-Aldrich, Steinheim, Germany, puriss.) were used as solvents. Chloroplatinic acid hexahydrate (ACS reagent, ≥37.50% Pt basis, Aldrich, St. Louis, MO, USA) was used for galvanic displacement.

### 3.2. Synthesis of Ni-Based Nanofibers

A common synthesis procedure was used for all the materials presented in this paper. The electrospinning solutions were prepared by first dissolving the carrier polymer in the solvents of choice, and, in a second stage, the nickel salt precursor was added; once a clear solution was obtained, it was transferred to a plastic syringe and fed to a linearly sliding electrospinning needle using Teflon tubes. The flow rate was regulated with a syringe pump. A Spraybase^®^ electrospinning system (Dublin, Ireland) equipped with a rotating drum and a 0–30 kV power supply was used in all of the experiments. 

Details of the optimized electrospinning solution/condition used are gathered in Table 1, while details of the thermal treatments are collected in Table 2.

### 3.3. Characterization of Ni-Based Nanofibers

The morphology of the electrospun materials before and after the different thermal treatments was characterized by SEM using a FEI Quanta FEG (Field Emission Gun) 200 (Hillsboro, OR, USA) equipped with EDS, and TEM using a JEOL 1200 EXII (Tokyo, Japan). 

XRD patterns of the Ni supports were recorded at room temperature in Bragg–Brentano configuration using a PANAlytical X’pert diffractometer (Almelo, Netherlands), equipped with a hybrid monochromator, operating with CuK_α_ radiation (λ = 1.541 Å), and using a step size of 0.1° 2θ in the 2θ domain from 20° to 80°.

Thermogravimetric analysis to determine the carbon content on the Ni/C composite fibers was performed in air up to 1000 °C (10 °C/min) using a Netzsch TG 439 thermobalance (Selb, Germany). 

The dynamic viscosity of the electrospinning solutions was measured with a shear rheometer (Paar Physica UDS 200, Graz, Austria) at RT.

### 3.4. Pt Deposition on to Nickel Nanofibres

Platinum was deposited on the nickel nanofibres from aqueous chloroplatinic acid hexahydrate solution using an approach described elsewhere [46]. At the end of the exchange process, the nanofibres were collected by filtration and carefully washed with ethanol and water. A complete exchange leads to 30% platinum loading.

### 3.5. Characterisation of Ni@Pt Nanofibres

The morphology of the Ni@Pt 1D materials has been characterized by TEM using a JEOL 2200FS (Tokyo, Japan) (Source: FEG) microscope operating at 200 kV equipped with a Charge Coupled Device camera Gatan USC (16 MP) (Pleasanton, CA, USA). For TEM analyses, samples were suspended in ethanol and sonicated before deposition on to carbon-coated copper grids. 

Electrochemical characterisation was carried out in a conventional three-electrode cell consisting of a glassy carbon rotating disk electrode (RDE) (working electrode, geometric area of 0.196 cm^2^), a reversible hydrogen electrode (reference electrode, RHE) and a platinum wire (counter electrode). A Pine bipotentiostat model AFCBP1 (Grove City, PA, USA) was used. All of the potential values are referred to the RHE. Inks were prepared dispersing 5 mg of catalysed support in 300 μL of isopropanol, 20 μL of water, and 15 μL of 5 wt % Nafion. These inks were then deposited onto the RDE surface with a micropipette to give a final Pt loading of ~100 μg·cm^−2^. Cyclic voltammetry was carried out at 100 mV/s in N_2_ saturated 0.1 M aqueous HClO_4_ and the electrochemical surface area of platinum was calculated by integrating the peak of hydrogen desorption from the Pt sites. ORR was conducted on an RDE in an oxygen saturated 0.1 M HClO_4_ aqueous electrolyte at increasing rotating speeds (400, 900, 1600, 2500 RPM) chosen in order to achieve equally spaced saturation currents (Koutecky-Levich).

## 4. Conclusions 

We investigated the effect of electrospinning and thermal treatment parameters on morphology and composition of 1D nickel, nickel oxide and nickel nitride. This systematic screening has given insights, enabling improved understanding of the formation process of hybrid and inorganic spun nanomaterials, leading to an informed choice of synthesis procedure by the possibility of tuning their structure and the related properties. The obtained nanofibers/ribbons/sponges of nickel, nickel oxide, nickel/carbon, and nickel nitride are promising candidates for application in catalysis as well as energy conversion and storage. As an example, the use of nickel electrospun materials as electrocatalyst supports for fuel cell electrodes after their surface modification by Pt galvanic displacement has been proofed in this work.

Considering the flexibility and good scalability of electrospinning, as well as the versatile applicability of Ni-based compounds, this work opens the way for the rational and large-scale production of metallic materials towards binary and multi-metallic systems with a widened application range.

## Figures and Tables

**Table 1:** Details of the optimized electrospinning parameters.

Carrier Polymer (wt %)	Nickel Precursor (wt %)	Solvent	Dynamic Viscosity (Pa·s)	Applied Voltage (kV)	Flow Rate (mL/h)	Target Distance (cm)
PVA (12)	Ni acetate (12)	Water	0.460	15	0.10	12
PVP (9-10-11)	Ni acetate (9-10-11)	Ethanol	0.086/0.092/0.126	10	1.00	12
PVP (10)	Ni acetate (10)	Ethanol:DMF 7:3 vol	0.064	10	1.00	12
PVP (11)	Ni nitrate (11)	Ethanol:water 1:1 wt	0.130	14	0.20	10
PVP (9)	Ni nitrate (9)	Ethanol:DMF 1:1 wt	0.071	20	0.25	12

PVA: polyvinylalcohol; PVP: polyvinylpyrrolidone; DMF: dimethylformamide.

**Table 2:** Details of the thermal treatments.

Procedure	Starting Material	Ramp (°C/min)	Dwell Time (min)	Dwell Temperature (°C)	Gas	Gas Flow (cc/h)	Obtained Phase
Calcination	Polymer + nickel salt	1	60	(I) 150	air	--	NiO
				(II) 250			
				(III) 600			
Reduction	NiO	5	30	250	H_2_	50	Ni
High temperature decomposition	Polymer + nickel salt	1	300	700	N_2_	50	Ni (C matrix)
Direct conversion into nitride	NiO	5	360	(I) 600	NH_3_	50	Ni_3_N
				(II) 250			
Conversion into nitride	Ni	5	0-60	250	NH_3_	50	Ni_3_N

## References

[1] Li Z., Li M., Bian Z., Kathiraser Y., Kawi S. (2016). Design of highly stable and selective core/yolk–shell nanocatalysts—A review. Appl. Catal. B.

[2] Liu C.J., Ye J., Jiang J., Pan Y. (2011). Progresses in the preparation of coke resistant Ni-based catalyst for steam and CO_2_ reforming of methane. ChemCatChem.

[3] Cai H., Liu L., Chen Q., Lu P., Dong J. (2016). Ni-polymer nanogel hybrid particles: A new strategy for hydrogen production from the hydrolysis of dimethylamine-borane and sodium borohydride. Energy.

[4] Cai W., Liu W., Han J., Wang A. (2016). Enhanced hydrogen production in microbial electrolysis cell with 3D self-assembly nickel foam-graphene cathode. Biosens. Bioelectron..

[5] Gong M., Wang D.Y., Chen C.C., Hwang B.J., Dai H. (2016). A mini review on nickel-based electrocatalysts for alkaline hydrogen evolution reaction. Nano Res..

[6] Xing Z., Li Q., Wang D., Yang X., Sun X. (2016). Self-supported nickel nitride as an efficient high-performance three-dimensional cathode for the alkaline hydrogen evolution reaction. Electrochim. Acta.

[7] Shalom M., Ressnig D., Yang X., Clavel G., Fellinger T.P., Antonietti M. (2015). Nickel nitride as an efficient electrocatalyst for water splitting. J. Mater. Chem. A.

[8] Lee M.J., Hong S.K., Choi B.H., Hwang H.J. (2016). Fabrication and performance of solid oxide fuel cell anodes from core-shell structured Ni/yttria-stabilized zirconia (YSZ) powders. Ceram. Int..

[9] Alia S.M., Larsen B.A., Pylypenko S., Cullen D.A., Diercks D.R., Neyerlin K.C., Kocha S.S., Pivovar B.S. (2014). Platinum-coated nickel nanowires as oxygen-reducing electrocatalysts. ACS Catal..

[10] Han B., Carlton C.E., Kongkanand A., Kukreja R.S., Theobald B.R., Gan L., O’Malley R., Strasser P., Wagner F.T., Shao-Horn Y. (2015). Record activity and stability of dealloyed bimetallic catalysts for proton exchange membrane fuel cells. Energy Environ. Sci..

[11] Tian X., Luo J., Nan H., Zou H., Chen R., Shu T., Li X., Li Y., Song H., Liao S. (2016). Transition Metal Nitride Coated with Atomic Layers of Pt as a Low-Cost, Highly Stable Electrocatalyst for the Oxygen Reduction Reaction. J. Am. Chem. Soc..

[12] Kuttiyiel K.A., Sasaki K., Choi Y., Su D., Liu P., Adzic R.R. (2012). Nitride Stabilized PtNi Core—Shell Nanocatalyst for high Oxygen Reduction Activity. Nano Lett..

[13] Zhuang Z., Giles S.A., Zheng J., Jenness G.R., Caratzoulas S., Vlachos D.G., Yan Y. (2016). Nickel supported on nitrogen-doped carbon nanotubes as hydrogen oxidation reaction catalyst in alkaline electrolyte. Nat. Commun..

[14] Cheng X., Ye K., Zhang D., Cheng K., Li Y., Wang B., Wang G., Cao D. (2015). Methanol electrooxidation on flexible multi-walled carbon nanotube-modified sponge-based nickel electrode. J. Solid State Electrochem..

[15] Mardanpour M.M., Yaghmaei S. (2016). Characterization of a microfluidic microbial fuel cell as a power generator based on a nickel electrode. Biosens. Bioelectron..

[16] Li W., Shi Y., Luo Y., Wang Y., Cai N. (2016). Carbon monoxide/carbon dioxide electrochemical conversion on patterned nickel electrodes operating in fuel cell and electrolysis cell modes. Int. J. Hydrogen Energy.

[17] Lim R.J., Xie M., Sk M.A., Lee J.M., Fisher A., Wang X., Lim K.H. (2014). A review on the electrochemical reduction of CO_2_ in fuel cells, metal electrodes and molecular catalysts. Catal. Today.

[18] Alia S.M., Yan Y., Pivovar B. (2014). Galvanic Displacement as a Route to Highly Active and Durable, Extended Surface Electrocatalysts. Catal. Sci. Technol..

[19] Alia S.M., Pylypenko S., Dameron A., Neyerlin K.C., Kocha S.S., Pivovar B.S. (2016). Oxidation of Platinum Nickel Nanowires to Improve Durability of Oxygen-Reducing Electrocatalysts. J. Electrochem. Soc..

[20] Zhang D., Wang B., Cao D., Ye K., Xu Y., Yin J., Cheng K., Wang G. (2014). N2H4 electrooxidation at negative potential on novel wearable nano-Ni-MWNTs-textile electrode. Mater. Sci. Eng. B.

[21] Guo F., Ye K., Du M., Cheng K., Gao Y., Wang G., Cao D. (2016). Nickel nanowire arrays electrode as an efficient catalyst for urea peroxide electro-oxidation in alkaline media. Electrochim. Acta.

[22] Muench F., Oezaslan M., Rauber M., Kaserer S., Fuchs A., Mankel E., Brötz J., Strasser P., Roth C., Ensinger W. (2013). Electroless synthesis of nanostructured nickel and nickel-boron tubes and their performance as unsupported ethanol electrooxidation catalysts. J. Power Sources.

[23] Pradhan B.K., Kyotani T., Tomita A. (1999). Nickel nanowires of 4 nm diameter in the cavity of carbon nanotubes. Chem. Commun..

[24] Tang S., Vongehr S., Ren H., Meng X. (2012). Diameter-controlled synthesis of polycrystalline nickel nanowires and their size dependent magnetic properties. CrystEngComm.

[25] Sun L., Chen Q., Tang Y., Xiong Y. (2007). Formation of one-dimensional nickel wires by chemical reduction of nickel ions under magnetic fields. Chem. Commun..

[26] Xia Z., Wen W. (2016). Synthesis of Nickel Nanowires with Tunable Characteristics. Nanomaterials.

[27] Wu H., Zhang R., Liu X., Lin D., Pan W. (2007). Electrospinning of Fe, Co, and Ni Nanofibers: Synthesis, Assembly, and Magnetic Properties. Chem. Mater..

[28] Ji Y., Zhang X., Zhu Y., Li B., Wang Y., Zhang J., Feng Y. (2013). Nickel nanofibers synthesized by the electrospinning method. Mater. Res. Bull..

[29] Barakat N.A.M., Kim B., Kim H.Y. (2009). Production of Smooth and Pure Nickel Metal Nanofibers by the Electrospinning Technique: Nanofibers Possess Splendid Magnetic Properties. J. Phys. Chem. C.

[30] Liu H., Wang X., He G., Lin Y., Wei J., Zheng J., Zheng G., Sun D. Electrospun nickel oxide nanofibers for gas sensor application. Proceedings of the 8th Annual IEEE International Conference on Nano/Micro Engineered and Molecular Systems, IEEE NEMS 2013.

[31] Macdonald T., Xu J., Elmas S., Mange Y., Skinner W., Xu H., Nann T. (2014). NiO Nanofibers as a Candidate for a Nanophotocathode. Nanomaterials.

[32] Cavaliere S., Subianto S., Savych I., Jones D.J., Rozière J. (2011). Electrospinning: Designed architectures for energy conversion and storage devices. Energy Environ. Sci..

[33] Khalil A., Hashaikeh R. (2014). Electrospinning of nickel oxide nanofibers: Process parameters and morphology control. Mater. Charact..

[34] Reneker D.H., Yarin A.L. (2008). Electrospinning jets and polymer nanofibers. Polymer (Guildf.).

[35] Koombhongse S., Liu W., Reneker D.H. (2001). Flat polymer ribbons and other shapes by electrospinning. J. Polym. Sci. Part B.

[36] Bandrowski J., Bickling C.R., Yang K.H., Hougen O.A. (1962). Kinetics of the reduction of nickel oxide by hydrogen. Chem. Eng. Sci..

[37] Janković B., Adnađević B., Mentus S. (2008). The kinetic study of temperature-programmed reduction of nickel oxide in hydrogen atmosphere. Chem. Eng. Sci..

[38] Richardson J.T., Scates R., Twigg M.V. (2003). X-ray diffraction study of nickel oxide reduction by hydrogen. Appl. Catal. A.

[39] Manukyan K.V., Avetisyan A.G., Shuck C.E., Chatilyan H.A., Rouvimov S., Kharatyan S.L., Mukasyan A.S. (2015). Nickel Oxide Reduction by Hydrogen: Kinetics and Structural Transformations. J. Phys. Chem. C.

[40] Afanasov I.M., Lebedev O.I., Kolozhvary B.A., Smirnov A.V., van Tendeloo G. (2011). Nickel/Carbon composite materials based on expanded graphite. New Carbon Mater..

[41] Li J., Liu E.H., Li W., Meng X.Y., Tan S.T. (2009). Nickel/carbon nanofibers composite electrodes as supercapacitors prepared by electrospinning. J. Alloys Compd..

[42] Balogun M.-S., Zeng Y., Qiu W., Luo Y., Onasanya A., Olaniyi T.K., Tong Y. (2016). Three-dimensional nickel nitride (Ni 3 N) nanosheets: Free standing and flexible electrodes for lithium ion batteries and supercapacitors. J. Mater. Chem. A.

[43] Soo Kang J., Park M.-A., Kim J.J.-Y., Ha Park S., Young Chung D., Yu S.-H., Kim J.J.-Y., Park J., Choi J.-W., Jae Lee K. (2015). Reactively sputtered nickel nitride as electrocatalytic counter electrode for dye- and quantum dot-sensitized solar cells. Sci. Rep..

[44] Baiker A., Maciejewski M. (1984). Formation and thermal stability of copper and nickel nitrides. J. Chem. Soc. Faraday Trans..

[45] Debe M.K. (2012). Electrocatalyst approaches and challenges for automotive fuel cells. Nature.

[46] Ercolano G., Cavaliere S., Jones D., Rozière J. (2015). Electrospun Ni nanofibres as Pt supports for PEMFC electrodes. ECS Trans..

